# Study protocol for the MIND-PD study: a randomized controlled trial to investigate clinical and biological effects of mindfulness-based cognitive therapy in people with Parkinson’s disease

**DOI:** 10.1186/s12883-024-03736-7

**Published:** 2024-06-25

**Authors:** Anouk van der Heide, Franziska Goltz, Nienke M. de Vries, Bastiaan R. Bloem, Anne E. Speckens, Rick C. Helmich

**Affiliations:** 1 Donders Institute for Brain, Cognition and Behaviour, Centre for Cognitive Neuroimaging, Radboud University, P.O. Box 9101, Nijmegen, 6500 HB The Netherlands; 2 Neurology department, Centre of Expertise for Parkinson & Movement Disorders, Radboud University Medical Centre, Nijmegen, The Netherlands; 3 Radboud University Medical Centre, Psychiatry department, Nijmegen, The Netherlands

**Keywords:** Parkinson’s disease, Mindfulness-based cognitive therapy, Depression, Anxiety, Disease progression, Non-pharmacological

## Abstract

**Background:**

People with Parkinson’s disease (PD) are very sensitive to the effects of stress. The prevalence of stress-related neuropsychiatric symptoms is high, and acute stress worsens motor symptoms. Animal studies suggest that chronic stress may accelerate disease progression, but evidence for this in humans is lacking. Mindfulness-based interventions (MBIs) train participants to focus on the present moment, on purpose and without judgement. Previous studies suggest that MBIs may alleviate stress and reduce depression and anxiety in PD. We aim to demonstrate the efficacy of Mindfulness-Based Cognitive Therapy (MBCT) as a non-pharmacologic treatment strategy for neuropsychiatric (and motor) symptoms in PD, and to identify the mechanisms underlying stress and stress reduction in PD.

**Methods:**

In a prospective randomized controlled trial (RCT), we investigate whether 8 weeks of MBCT, as compared to care as usual, can reduce symptoms of anxiety and depression in people with PD. We aim to include 124 PD patients, who experience mild-moderate symptoms of anxiety and depression, are eligible for magnetic resonance imaging (MRI) and naïve to mindfulness, and who have a disease duration ≤ 10 years. Every participant is followed for 12 months. Clinical and biochemical assessments take place at baseline (T0), after 2 months (T1), and after 12 months (T2); MRI assessments take place at T0 and T2. Our primary outcome is the total score on the Hospital Anxiety and Depression Scale (HADS) at T1, while correcting for the HADS score at T0, age, and gender. Beyond testing the effects of MBCT on symptoms of anxiety and depression in PD, we explore whether MBCT: (1) has an effect on motor symptom severity, (2) influences cerebral and biochemical markers of stress, and (3) leads to a change in biomarkers of PD progression.

**Discussion:**

MIND-PD is one of the first RCTs with a 1-year follow-up to investigate the effects of MBCT on symptoms of anxiety and depression in PD, and to explore possible mechanisms underlying stress and stress reduction in PD. Insight into these mechanisms can pave the way to new treatment methods in the future.

**Trial registration:**

ClinicalTrials.gov, NCT05779137. Registered on 12 January 2023.

**Supplementary Information:**

The online version contains supplementary material available at 10.1186/s12883-024-03736-7.

## Introduction

### Background and rationale

Parkinson’s disease (PD) is the second most common neurodegenerative disorder, and one of the fastest-growing brain disorders globally [1]. Clinically, PD is characterized by motor slowing (bradykinesia), stiffness (rigidity) and resting tremor, and it is pathologically linked to nigro-striatal dopaminergic cell loss [2]. Increasing evidence suggests that stress plays a significant role in the development and progression of PD. Degeneration of the noradrenergic stress system has been linked to the high prevalence of severe stress symptoms, including depression and anxiety, in people with PD [3]. Also, acute stress significantly aggravates motor symptoms [4], and animal models suggest that chronic stress may have detrimental long-term consequences by accelerating disease progression [5, 6].

Currently, there is no cure for PD, and no treatments to slow down disease progression. Therefore, the development of new and effective treatment strategies is crucial. Given the evident symptomatic effects of stress on PD, as well as its potential pathophysiologic link to the disease, treatments targeting the alleviation of stress are promising [7]. Although the evidence for effective stress reduction in PD is still scarce, recent findings suggest that mindfulness-based interventions (MBIs) may be effective in achieving short- as well as long-term stress reduction in people with PD, presumably by improving coping mechanisms [8]. Mindfulness is the trainable capacity to experience the present moment, on purpose and without judgment, while accepting experienced emotions [9]. MBIs, such as mindfulness-based cognitive therapy (MBCT), have been shown to reduce symptoms of depression and anxiety in various psychiatric and somatic conditions, such as major depressive disorder, cancer, and multiple sclerosis [10, 11]. In PD, several trials investigated the effects of MBIs, overall showing positive effects on stress symptoms such as depression, anxiety, and quality of life [12–14]. Intriguingly, some studies also suggest a reduction of motor symptom severity following an MBI [12, 13]. Structural changes in the brain have even been observed [8, 15]. A large online survey in 5,000 people with PD further showed that mindfulness users report positive effects of mindfulness on symptoms of anxiety and depression, and that the magnitude of this effect was associated with the amount of mindfulness practice [4].

Although this prior work is promising, the current evidence has substantial limitations. To date, only one sufficiently-powered trial investigated the effect of an MBI on PD symptoms, suggesting positive effects of mindfulness practice on both motor and non-motor symptoms [13]. However, the effects of MBIs on motor impairments are not consistent across studies [12, 13, 16, 17]. Also, long-term effects (beyond 3 months) of MBIs in PD have not been investigated. Finally, the mechanisms underlying the benefits of such interventions remain unknown. In this study, we therefore use a randomized controlled trial with a longer term follow-up (12 months) to test whether MBCT reduces symptoms of anxiety and depression and improves motor symptoms in PD. We will also explore the possible cerebral and biochemical mechanisms underlying MBCT. These insights can pave the way for developing new, mechanism-based interventions, and can help to uncover how acute and chronic stress affect PD.

### Objectives

The primary objective of this study is to investigate whether MBCT in addition to care as usual (CAU), compared to CAU only, can reduce symptoms of anxiety and depression in people with PD, who experience mild to moderate levels of such symptoms at baseline. We consider anxiety and depression as severe stress symptoms and indicators of a maladaptive stress-response, resulting from enhanced or chronic levels of stress [7]. Our secondary objectives are to investigate whether the provided intervention (1) has an effect on motor symptom severity, (2) influences cerebral and biochemical markers of stress, and (3) changes biomarkers of PD progression.

### Trial design

The MIND-PD study is a prospective multicenter randomized controlled trial with an intervention (MBCT + CAU) and a control arm (CAU) (1:1 ratio). The study duration per participant is 1 year and comprises three measurement timepoints: baseline (T0), two months after baseline (T1; post-intervention for MBCT group), and 12 months after baseline (T2). Symptoms of anxiety and depression (as measured with the Hospital Anxiety and Depression scale [HADS]) at T1 is the primary outcome. The study protocol has been approved by the local ethical review board (METC Oost-Nederland) and is registered under 2022–15931.

## Methods: participants, interventions, randomization, procedures, and outcomes

### Study setting

This study will be performed at the Donders Centre for Cognitive Neuroimaging (DCCN) and the Radboud university medical center (Radboudumc) in Nijmegen. The Radboudumc is the sponsor of the study and the group-MBCT will be provided at the Radboudumc Center for Mindfulness. Acquisition of all outcomes (Magnetic Resonance Imaging (MRI), clinical assessments, biochemical material) will be performed at the DCCN.

### Study population

The study population consists of people with a diagnosis of PD, made by a movement disorders specialist according to the Movement Disorders Society (MDS) criteria [18], and with a disease duration ≤ 10 years (defined as time since diagnosis made by a neurologist). Additional inclusion criteria are (1) mild-moderate symptoms of depression and anxiety (HADS-total score > 10 points) and (2) ability to read and understand the Dutch language. A subject who meets any of the following criteria will be excluded from participation:

1. Severe neurological or psychiatric comorbidities (e.g. psychosis or suicidality).
2. Contraindications for MRI (e.g., brain surgery in medical history, claustrophobia, an active implant, epilepsy, pregnancy, and/or metal objects in the upper body that are incompatible with MRI).
3. Moderate to severe head tremor (to avoid artifacts caused by extensive head motion during MRI scanning).
4. Cognitive dysfunction (clinical diagnosis of dementia, or a score of ≤ 20 on the Montreal Cognitive Assessment (MoCA), which will be assessed at T0).
5. Previous participation in mindfulness-based stress reduction or MBCT program (> 4 sessions) in recent years.

### Interventions

#### MBCT

The 8-week curriculum of the group-based intervention is in line with the MBCT program described by Segal and colleagues [19]. For the purpose of this study, the content of the self-study materials was slightly adjusted to address PD-related issues and difficulties explicitly. For example, materials specifically discuss mindful living with physical restrictions associated with PD, and describe how to deal with lifestyle changes imposed by the disease. The intervention is provided to groups of 8–10 patients, which are a combination of both MIND-PD study participants as well as people with other somatic illnesses, who are referred to the training otherwise. The MBCT consists of eight weekly sessions of 2.5 h and one 4-hour silence day between the 6th and 7th session. The sessions include meditation exercises (body scan, sitting meditation, gentle movement exercises, three-minute breathing space, daily activities with attention), psychoeducation and group discussions. Psychoeducation includes information about cognitive techniques, like monitoring and scheduling of events, as well as identification of negative automatic thoughts. In addition, all participants are encouraged to perform daily practice assignments at home for about 30–45 min per day, mainly consisting of meditation exercises. Teachers of the MBCT fulfill the advanced criteria of the Association of Mindfulness Based Teachers in the Netherlands and are registered in the Dutch register for mindfulness trainers (https://www.mindfulnessregister.nl/). These criteria are in line with the UK Mindfulness-Based Teacher Trainer Network Good Practice Guidelines for teaching mindfulness-based courses [20]. During each MBCT course, two supervision meetings are held between the mindfulness teacher(s) and the intervention supervisor (AS). In addition, two MBCT courses per teacher will be video-recorded in order to assess teacher competence and protocol adherence using the Mindfulness-Based Interventions-Teachers Assessment Criteria [21]. To monitor adherence of the participants to the intervention, the Mindfulness Adherence Questionnaire [22] will be administered during MBCT sessions 2–8, as well as every 2 months after completion of the intervention. To further encourage mindfulness adherence in the course of the follow-up period, participants will receive bi-monthly newsletters providing updates about the trial, as well as motivational videos and materials discussing mindfulness and its potential benefits. The MBCT intervention is an add-on treatment; participants remain on their usual (dopaminergic) treatment during the intervention. While participating in the study, patients are asked to refrain from starting any new treatments apart from the study intervention, unless prescribed by their doctor. Health care use in the intervention group is monitored as part of the home questionnaires at T0, T1 and T2, as well as 6 months after T0 by means of the Treatment Inventory of Costs in Patients with psychiatric disorders (TIC-P, item 1–14) [23]. At the same timepoints, relevant lifestyle factors are monitored in the home questionnaires.

#### CAU

Participants in the CAU arm do not receive any add-on intervention and will maintain their usual treatment. To retain commitment with the trial, and to ensure an equal amount of contact with the research personnel in both groups, participants in the CAU group will also receive bi-monthly newsletters with updates about the trial, as well as materials that provide information about PD related topics, such as gastrointestinal problems. Information in the newsletters provided to this group will not be related to mindfulness or other lifestyle interventions. Participants in the CAU arm are asked to refrain from starting any new treatments in the course of the study, unless prescribed by their doctor. Health care use is monitored by the TIC-P as described above, as well as relevant lifestyle factors.

### Randomization and blinding

Participants are randomized using a Good Clinical Practice-compliant system (CastorEDC; https://www.castoredc.com/), which employs stratified, variable block randomization. After their baseline visit, participants are randomized in a 1:1 ratio to the treatment (MBCT + CAU) or control (CAU) group. The randomization sequence is determined by variable block sizes (2,4) and is stratified by gender (female, male, or other) and age (< 65 years old, and ≥ 65 years old). Group allocation is communicated by the coordinating researcher (FG) after T0. Participants are aware of the study design and are therefore not blinded to their study condition. Data acquisition will be performed by the coordinating researcher, hence data acquisition is not blinded. Outcome measures involving structural and functional neuroimaging, as well as other biological markers or self-report questionnaires are unlikely to be influenced by the (unblinded) researcher. To enable post-hoc ratings of disease severity (score on MDS Unified Parkinson’s Disease Rating Scale (UPDRS) III) by an independent and blinded researcher, motor performance will be video-recorded.

### Procedures

**Fig. 1:**
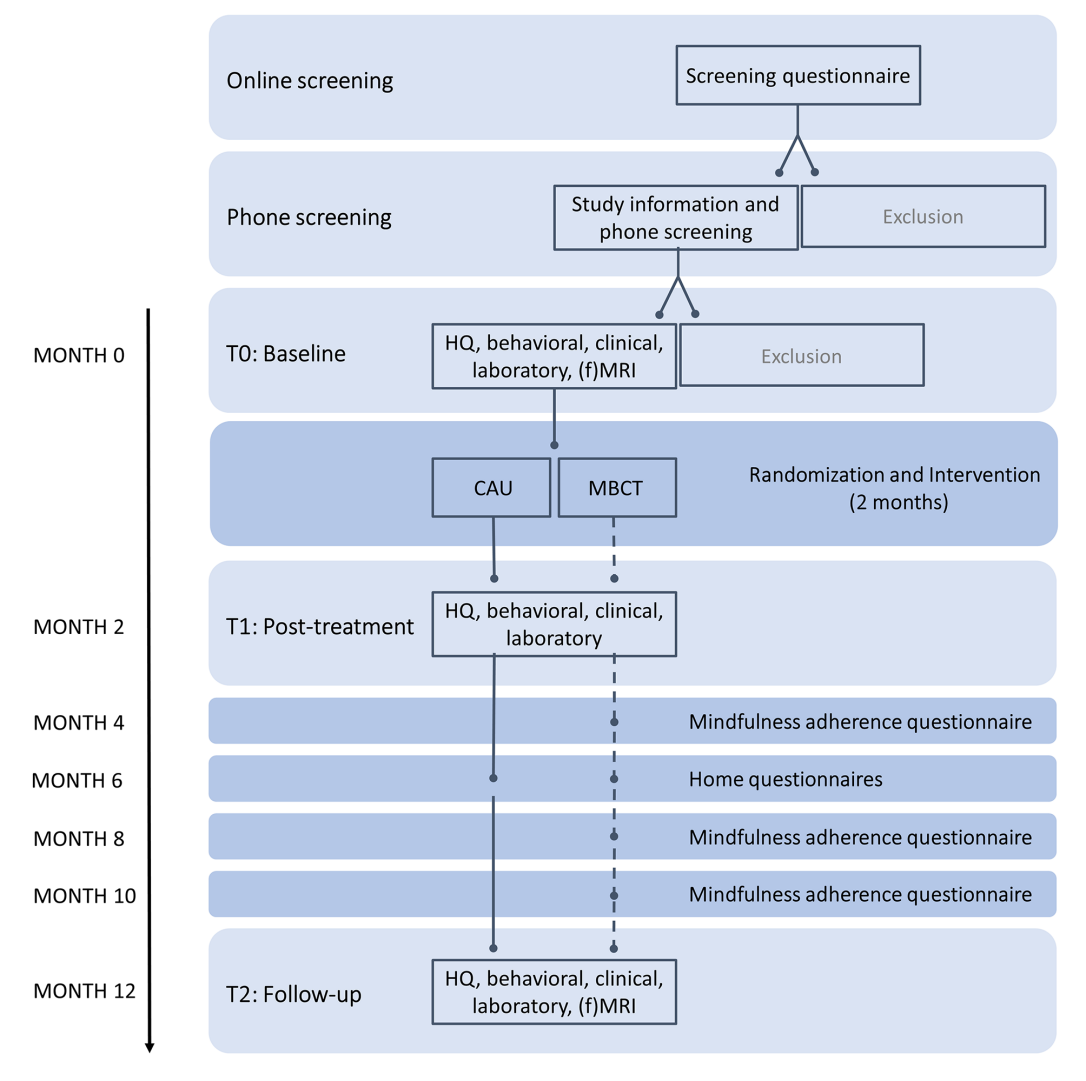
Recruitment and assessment flow of MIND-PD. Study participants are screened for eligibility prior to their baseline visit (T0). After T0, participants are randomized into one of the study arms (MBCT + CAU or CAU). T1 and T2 assessments take place at month 2 and 12, respectively. CAU = care as usual; MBCT = mindfulness-based cognitive therapy; HQ = home questionnaires; fMRI = functional magnetic resonance imaging

See Fig. 1 for the general flow of the study.

#### Recruitment

Patients will be recruited via three sources:

1. Attending clinicians at the outpatient clinic of the Radboudumc are asked to preselect patients they see during consultation or during a multidisciplinary meeting. In addition, we will recruit patients treated by neurologists in surrounding hospitals (for example in Den Bosch, Arnhem and Tiel). Attending neurologists will give information about the study and initial inclusion criteria. Only patients that show interest will receive more information about the study.
2. We will contact patients that gave permission to be approached for new scientific studies about PD during their participation in previous studies in our center.
3. The online platform ParkinsonNEXT (https://www.parkinsonnext.nl*)* will be used, through which people with PD can easily sign up for research projects. The ParkinsonNEXT website contains a webpage with general information about our project, including background information and the possibility to register directly. In addition, we will send an open invitation to participate in our study via ParkinsonNEXT to all registered patients living close to Nijmegen.

If people are interested to participate, the researchers will provide them with information and will invite them to complete the online screening questionnaires to check their eligibility. If the patient is interested and eligible, they will receive the participant information letter. A week later, the researcher will call to provide more information and answer remaining questions if necessary. Afterwards, the patient can give verbal consent and the first visit is planned. During the first visit, the procedure, details of the study and obligations of the participant and the clinical research team will be clearly explained to all patients once more, and written informed consent is obtained. Only subjects who can give legal consent will be included. Participants will be recruited in multiple study cohorts of 10–16 patients each. Accordingly, groups of ~ 5–8 patients will simultaneously participate in each study arm (exact numbers depend on the randomization algorithm).

#### Baseline and follow-up assessment

At baseline (T0) and follow-up (T2), participants will visit the DCCN in a practically defined OFF-state (last levodopa intake ≥ 12 h prior to testing, 24 h for delayed release levodopa and dopaminergic agonists, 48 h for delayed release dopamine agonists) for a total of about 4 h, scheduled between 08:00 h and 15:00 h. Clinical, laboratory and neuroimaging assessments will be performed during the lab visit (see Table 1 for an overview of all outcome measures). All baseline assessments will take place within 8 weeks prior to the start of an intervention cohort; questionnaires will be filled out at home ON medication, within 1 week prior to the lab visit. The behavioral assessment will be completed within 1 week after the lab visit (ON medication). Assessments will typically be performed in the following order: neurological assessment and cognitive abilities (MDS-UPDRS, MoCA), laboratory assessments (blood, scalp hair), (f)MRI, additional motor symptom assessments (tremor, bradykinesia). Follow-up assessments will take place at least 12 months after baseline. Procedures during the T2 lab visit will fully match those of the participants’ baseline assessment.

#### Post-treatment assessment

Post-treatment visits (T1) will take place at least 2 months after baseline. To prevent any structural group differences with regards to the T0-T1 time interval, both study arms will be assessed after the MBCT training of the respective cohort has concluded. At T1, participants will visit the DCCN for a total of about 1.5 h in practically defined OFF-state, scheduled between 08:00 h and 12:00 h. Questionnaires and the behavioral assessment will be acquired as described above. During the T1 visit, assessments will typically be performed in the following order: neurological assessment and cognitive abilities (MDS-UPDRS, MoCA), additional motor symptom assessments (tremor, bradykinesia), laboratory assessments (blood, scalp hair).

### Primary outcome

The primary endpoint of this trial are symptoms of anxiety and depression at T1 (post-intervention), as measured by the total score (range 0–42) on the Hospital Anxiety and Depression Scale (HADS). Higher scores indicate more symptoms. The HADS is a 14-item self-report questionnaire, which has been validated as a reliable, consistent instrument in people with PD [24]. Previously, the HADS has been used as primary outcome measure in the largest MBI-RCT in PD to date [13], and RCTs investigating the effects of MBCT in other somatic illnesses have implemented the HADS as primary outcome as well [25, 26]. Good psychometric properties of the Dutch HADS-total have been demonstrated in non-PD samples (Cronbach’s α = 0.82–0.90; [27]).

### Secondary outcomes

An overview of all study outcomes can be found in Table 1.

**Table 1:** Overview of all study outcomes

Assessment type	HQ	Visit	HQ	Visit		HQ			HQ	Visit
Study timepoint	T0	T0	T1	T1					T2	T2
Months after baseline	0	0	2	2	4	6	8	10	12	12
**Clinical assessments**										
Motor and non-motor symptoms (MDS-UPDRS III, IV, Ia)		X		X						X
Tremor severity (Accelerometry)		X		X						X
Bradykinesia (Finger tapping test)		X		X						X
Cognitive abilities (MoCA)		X		X						X
**Behavioral assessments**										
Controllability update task		X		X						X
**Laboratory measures**										
Scalp hair cortisol		X		X						X
High sensitivity C-reactive protein		X		X						X
Salivary cortisol		X								X
**Neuroimaging ((f)MRI)**										
Structural scan (T1 MPRAGE)		X								X
Neuromelanin sensitive scan (T1 TSE)		X								X
Diffusion weighted imaging (DTI)		X								X
Resting state fMRI (MB-ME3) before and after stress induction		X								X
**Questionnaires**										
Anxiety and depression (HADS)^1^	X		X			X			X	
Perceived stress (PSS)	X		X			X			X	
Rumination (RRS)	X		X			X			X	
Self-compassion (SCS)	X		X			X			X	
Self-efficacy (GSES)	X		X			X			X	
Positive appraisal style (PASS)	X		X			X			X	
Mindfulness skills (FFMQ)	X		X			X			X	
Perceived social support (F-SozU K6)	X		X			X			X	
Quality of life (PDQ-39)	X		X			X			X	
Motor and non-motor experiences of daily living (MDS-UPDRS Ib, II)	X		X			X			X	
**MBCT arm only**										
Mindfulness adherence (MAQ)			X		X	X	X	X	X	

^1^ Primary outcome

Study timepoints are at baseline (T0) two months after baseline (T1) and 12 months after baseline (T2). HQ = home questionnaire.

#### Clinical assessments

*Motor impairments and -complications* will be assessed by the MDS-UPDRS III and IV [28]; global non-motor symptoms will be assessed by the MDS-UPDRS Ia.

*Tremor severity* will be measured with a tri-axial accelerometer (Brain Products, sampling frequency 5 kHz, range: ±2 g, sensitivity: 1450 mV/g ± 5%) placed in the middle of the dorsum of the most affected hand. Tremor amplitude and -frequency will be assessed during four conditions: *rest* (participant seated, forearms supported, wrists unrestricted), *posture* (both arms lifted and stretched forward), *action* (slowly bending wrist up and down) and *cognitive coactivation* (forearms supported, wrists unrestricted, while performing a mental arithmetic task).

A previously reported keyboard finger tapping test [29] will be used to quantify *bradykinesia*. The test consists of two tapping tasks (alternating between the “M/N” keys for 30 s and between the “P/Q” keys 20 times as quickly as possible) and will be performed for 12 trials in total. The order of the tasks (“M/N” vs. “P/Q”) will be randomized. Patients will perform the test with the index finger of their most affected hand.

*Cognitive abilities* will be assessed by means of the MoCA [30]. The Dutch versions 7.1, 8.2 and 8.3 will be used at the three measurement timepoints, respectively. Test-retest reliability between alternative forms of the Dutch MoCA is good-excellent (ICC = 0.64–0.82) [31].

#### Behavioral assessments

To assess controllability perception, participants will perform a previously reported *controllability update task* [32]. During this online computer task, patients are exposed to two different environments, in which they either experience control (outcomes are dependent on their actions), or they experience lack of control (outcomes are independent on their actions). By exploring the environment and the outcomes of their actions, patients can experience in which environment they are. Subsequent to exploration, patients are asked to predict outcomes of their actions, while their answers and response patterns are monitored. By fitting two statistical learning models to those response patterns, it can be determined whether a person experiences their environment as rather controllable or uncontrollable. The two models represent an ‘actor mode’ (actions determine outcome) and a ‘spectator mode’ (actions do not matter in the outcome). The relative explanatory value of the two models gives insight into a patient’s bias for either mode and hence their ability to correctly identify environments in which their actions do or do not matter.

#### Laboratory measurements

*Scalp hair cortisol* will be collected to assess chronic stress in the preceding two months. Hair will be collected of subjects with a hair length of at least 3 centimeters. Analysis will be performed by the Radboudumc laboratory for diagnostics (RLD).

*High sensitivity C-reactive protein (hsCRP)* will be collected to assess inflammatory tone. Within two hours after blood drawing, samples will be centrifuged and hsCRP plasma levels will be extracted by the RLD. Plasma and whole blood will be stored at -80 °C for the remaining duration of the trial.

*Salivary cortisol* will be collected to assess the endocrine response to a laboratory stress-induction (see *socially evaluated cold pressor task* below). Four salivette tubes will be administered at T0 and T2. Specifically, saliva samples are collected 30–60 min prior to stress-induction, immediately after stress-induction, 30 min after stress-induction and 60 min after stress-induction. Saliva samples will be frozen and stored at -20℃ until analysis. After thawing, samples will be centrifuged at 3,000 rpm for 5 min. Salivary concentrations will be measured using commercially available chemiluminescence immunoassay with high sensitivity (Tecan - IBL International, Hamburg, Germany; catalogue number R62111).

#### Neuroimaging

All subjects will be scanned on a SIEMENS Prisma or Prismafit 3T MRI system. Scanner type will be consistent within subjects. The following scans will be acquired:

- *T1 weighted MPRAGE structural image* (TR: 2300ms, TE: 3.03ms, FA: 8 deg, TI: 1100ms, voxel size: 1.0 × 1.0 × 1.0 mm, FOV: 256 mm; acquisition time: 5m21s).
- Neuromelanin-sensitive *turbo spin echo T1 weighted image* to quantify structural integrity of the substantia nigra and locus coeruleus (TR: 890ms, TE: 13ms, FA: 120 deg, voxel size: 0.2 × 0.2 × 3.0 mm, FOV: 220 mm; acquisition time: 6m48s).
- *Diffusion weighted image* to quantify free water volume in the substantia nigra (TR: 3000ms, TE: 74.40ms, FA: 90 deg, voxel size: 2.0 × 2.0 × 2.0 mm, acceleration factor: 3, diffusion directions: 104, B-values: 1000 and 2000s/mm^2^, FOV: 210 mm; acquisition time: 5m30s).
- Resting-state BOLD EPI (functional MRI) images (eyes open, fixation cross) will be acquired twice: before and after stress induction (multi-band 3 multi-echo 3, TR: 1500ms, TE: 13.4ms / 34.8ms / 56.2ms, FA: 75 deg, voxel size: 2.5 × 2.5 × 2.5 mm, FOV: 210 mm, 400 volumes; acquisition time: 10m18s).

Participants’ stress-reaction will be investigated by means of the *socially evaluated cold pressor task* (SECPT) [33]. Resting-state functional MRI will be acquired before and after stress-induction (see imaging details above*)*. A researcher unknown to the participant will enter the room to perform the stress-induction task. The participant is asked to immerse their foot in a box of cold water (1.8–2.2℃) and to try keeping it in for three minutes, unless it is unbearable to continue. The participant is told that their facial expressions are evaluated during the task. The researcher is instructed to refrain from giving any positive feedback during the task and to try to keep a neutral facial expression. The second part of the stress-induction continues once the cold pressor test is over and the participant’s foot is covered with a towel. During the second part of the task, the participant is asked to alternatively cite the alphabet from the back and front (Z-A-Y-B-X-C etc.), as fast and as accurately as possible. If they are too slow, the researcher asks them to speed up. If they are incorrect, the researcher asks them to start from the beginning. This task lasts 3 min. Subjective stress levels will be collected before and after stress-induction on a 10-point scale [1 (not stressed at all) − 10 (very stressed)].

#### Questionnaires

All questionnaires will be assessed online via CastorEDC.

*The Perceived Stress Scale* (PSS), a questionnaire consisting of 10 items, is used to measure perceived stress (range 0–40). It assesses how unpredictable, uncontrollable, and overloaded respondents experience their lives in the past month. Responses are given on a 5-point Likert scale. Studies have demonstrated good reliability of the PSS (Cronbach’s α = 0.78) [34].

The *Ruminative Response Scale* (RRS) is a 22-item questionnaire that measures the tendency to use ruminative thinking when being in a negative mood (range 22–88). Items are rated on a 4-point Likert scale. The Dutch RRS has been shown to have excellent internal consistency (Cronbach’s α = 0.90) [35].

The *Self-Compassion Scale Short Form* (SCS– SF) consisting of 12 items (range 12–84) is used to measure self-compassion on six scales: self-kindness, self-judgment, common humanity, isolation, mindfulness and over-identification. Items are scored on a 7-point Likert scale. A total self-compassion score is computed by reversing the negative subscale items and combining the subscale scores. Higher scores indicate more self-compassion. The psychometric properties of the Dutch SCS-SF have been shown to be good (Cronbach’s α = 0.87) [36].

We use a Dutch adaptation of the *General Self-Efficacy Scale* (GSES) [37].The GSES is a 10-item questionnaire (range 10–40) to measure general sense of perceived self-efficacy, which is thought to predict the ability to cope with adversity. Good reliability of the GSES has been reported (Cronbach’s α = 0.75–0.90) [38].

Positive appraisal style, the tendency to appraise potentially threatening situations in a positive way, has been proposed to be an important resilience mechanism, and therefore offers a potential target for stress-alleviating approaches [39]. The *Positive Appraisal Style Scale (PASS)*, based on the German 14 item PASS-process described by Petri-Romao and colleagues [40] will be used to measure positive appraisal style.

The *Five Facet Mindfulness Questionnaire* (FFMQ) is used to measure mindfulness skills [41]. The FFMQ consists of 39 items divided into 5 facets: observing, describing, acting with awareness, non-judging and non-reactivity. Each item has 5 answer options, where higher scores reflect more mindfulness skills (range 39–195). All facets of the Dutch version in a (non-PD) sample with depressive symptomatology have been shown to be reliable (Cronbach’s α = 0.73–0.91) [42].

Perceived social support will be measured using a Dutch translation of the brief *Perceived Social Support Questionnaire* (F-SozU K-6) [43], a 6 item questionnaire measuring perceived and anticipated social support on a 5-point Likert scale (range 6–30). The German F-SozU K-6 has been shown to have high internal consistency (Cronbach’s α = 0.90).

We assess quality of life using the *Parkinson’s disease questionnaire 39* (PDQ-39). This questionnaire consists of 39 items in 8 domains: mobility (10 items), activities of daily living (ADL, 6 items), emotional well-being (6 items), stigma (4 items), social support (3 items), cognitions (4 items), communication (3 items) and bodily discomfort (3 items). All items are scored on a 5-point Likert scale. Items are first summed and then linearly transformed to a 0–100 scale. The internal consistency has been shown to be high for all but one domain (Cronbach’s α = 0.71–0.91 for 7 of the 8 domains, and 0.59 for ‘bodily discomfort’) [44].

*Non-motor and motor complications* during daily living will be assessed by the MDS-UPDRS Ib and II [28], a 20 item questionnaire. PD related impairments are rated on a 5 point scale (0: normal – 4: severe).

Finally, adherence to the mindfulness intervention will be assessed using a Dutch version of the 12-item *Mindfulness Adherence Questionnaire* (MAQ) [22]. Items are scored on a 7-point Likert-scale. This questionnaire measures the duration and frequency with which both formal and informal mindfulness exercises are performed; higher scores reflect a higher practice frequency for a specific type of exercise.

### Power and sample size estimate

The primary outcome is the HADS-total score at T1 (post-intervention). Based on a recent large RCT analyzing the effects of an MBI on the HADS in people with PD [13], we expect a moderate effect size (*d* = 0.48). This is also in accordance with a recent review of 44 meta-analyses investigating the effects of MBIs in different clinical populations, reporting effect sizes between 0.45 and 0.75 after an MBI when compared to care as usual [10]. Considering an effect size of 0.48, a comparison of two groups of 70 subjects would yield 80% power with a two-sided alpha of 0.05. We corrected our power for correlations between the covariate (HADS at T0) and outcome measure (HADS at T1), given our planned statistical model where we will include HADS at T0 as a covariate (see below). Assuming a conservative correlation (r) of 0.5 between these two measures, a sample size of *n* = 53 [70 × (1-r^2^)] patients per group is required [45]. Considering a potential drop-out of 15%, we will include 62 individuals per group. This sample size allows us to detect an absolute difference in HADS score of 3.1, which exceeds the minimal clinically important difference for the HADS in people with PD [24]. This will also allow us to demonstrate group effects with an effect size of > 0.50 for our secondary outcomes [46, 47].

## Methods: data management and statistical analyses

### Data management & confidentiality

All personal data will be handled in compliance with the EU General Data Protection Regulation. In all documents, subjects will be identified by an identification code to maintain pseudonymity (pseudonymisation entails to work with an identifier (a key-file) which allows the link between individuals and their data). The pseudonymisation key-file will be stored in an access restricted folder on the Donders file server, separately from the experimental data. Any personal data will be deleted as soon as it is no longer needed. Questionnaires and experimental data will be managed in CastorEDC, a username and password protected data capture system. All data will be stored on the Donders Institute infrastructure and archived on the Donders repository for 15 years upon study completion. Biochemical data will be destroyed upon completion of the trial. A data management plan further describing the location and access of study data, coding methods, and archiving of data has been approved by the Board of Directors of the Radboudumc.

### Statistical analyses

All analyses will be performed on an intention to treat basis. Longitudinal analysis (> 2 timepoints) will be performed with linear mixed models. Missing data will be imputed if necessary and possible.

#### Primary outcome

To determine whether MBCT can reduce symptoms of anxiety and depression in people with PD, our *primary analysis* will test the effects of GROUP (MBCT vs. CAU) on the HADS-total after treatment (T1). Specifically, an analysis of covariance (ANCOVA) will be used with HADS score at T1 as dependent variable and GROUP as fixed factor. Age at T0, gender and HADS at T0 will serve as covariates. *Secondary analyses* will explore the consolidation of treatment effects across TIME (T0 vs. T1 vs. T2) using mixed models (fixed effects: time, group, time*group; random intercept for SUBJECT). In case of a significant intraclass correlation coefficient, we will add COHORT as a random effect to account for clustering within intervention cohorts. Potential moderators of the treatment effect, such as cognitive impairment (MoCA at T0), disease severity (defined as time since diagnosis made by a neurologist), or age may be explored to identify subgroups of patients that particularly benefit from the intervention. Also, mediators of the effect, such as mindfulness skills (FFMQ), self-compassion (SCS), or rumination (RRS) may be tested, to explore psychological mechanisms underlying the effect of MBCT on symptoms of anxiety and depression.

#### Clinical assessments

To explore whether MBCT has an effect on motor symptom severity, we will test the effect of TIME (T0 vs. T1 vs. T2) and GROUP (MBCT vs. CAU) on MDS-UPDRS III scores in a linear mixed model (LMM) for repeated measures. We will also explore tremor amplitude as a function of GROUP (MBCT vs. CAU), TIME (T0 vs. T1 vs. T2) and CONDITION (rest vs. cognitive coactivation) using LMM. Similarly, bradykinesia scores derived by the key tapping task will be investigated as a function of GROUP and TIME. Further explorative analyses will test the effects of GROUP and TIME on cognitive abilities (MoCA), as well as self-reported psychological wellbeing and mindfulness skills (perceived stress, rumination, self-compassion, self-efficacy, positive appraisal style, mindfulness skills, social support and quality of life).

#### Behavioral assessments

We will use computational modeling to establish whether an actor or spectator model best explains a patient’s response patterns in a controllability update task. Specifically, from the behavioral data, a parameter is derived that captures the relative explanatory value of the actor and spectator model in the data. If the explanatory value of the actor model is larger than the spectator model, an environment is perceived as controllable. We will compare this parameter as a function of GROUP (MBCT vs. CAU) and TIME (T0 vs. T1 vs. T2).

#### Laboratory measurements

To explore whether MBCT influences biochemical markers of stress, the area under the curve with respect to increase [48] of *salivary cortisol* secretion pre- until 60 min post stress induction will be assessed as a function of TIME (T0 vs. T2) and GROUP (MBCT vs. CAU), while considering relevant covariates, such as age and gender. The predictive value of e.g. HADS at T0 and disease duration on the cortisol response will be explored using LMM. Similarly, changes in *hair cortisol levels* will be compared between T0, T1 and T2, and GROUP by means of LMM. To test the effect of MBCT on inflammatory tone (*hs-CRP*), causal mediation analysis techniques will be used to decompose causal effects of stress on neurodegeneration into four components: mediation (by inflammation) only, interaction (with inflammation) only, both, or neither.

#### Neuroimaging

Neuroimaging data will be analyzed using neuroimaging software packages, such as FMRIB Software Library (FSL; https://fsl.fmrib.ox.ac.uk/fsl/) and statistical parametric mapping (SPM), as well as custom analysis scripts. Data will be pre-processed to remove motion and imaging artefacts; general linear models will be used to further clean the data from physiological noise and other nuisance signals. To test the effects of MBCT on cerebral markers of disease progression, we will explore the structural integrity of the substantia nigra and locus coeruleus as a function of TIME (T0 vs. T2) and GROUP (MBCT vs. CAU). Similarly, free water volume in the substantia nigra will be estimated by running a bi-tensor model on the diffusion weighted images [49]. Estimates will be compared between TIME and GROUP. The effects of MBCT on the cerebral stress response will be tested as follows: Subject specific connectivity estimates of relevant resting-state networks will be generated by first performing spatial group independent component analysis (ICA) in FSL-MELODIC (Multivariate Exploratory Linear Optimized Decomposition; www.fmrib.ox.ac.uk/fsl), and subsequently performing dual regression [50]. Stress-related networks (default mode network, salience network, executive control network) will be selected based on previous literature [51]. Resulting network connectivity estimates will be analyzed in a mixed-effects model with GROUP (MBCT vs. CAU), TIME (T0 vs. T2), STRESS (PRE vs. POST) and relevant interactions as fixed effects; a random intercept for SUBJECT will be included.

## Methods: monitoring, ethics and dissemination

### Monitoring

As this study has a negligible risk classification, it does not require a data monitoring committee. The study will be monitored by an independent, certified monitor according to the Netherlands Federation of University Medical Centres guidelines for monitoring of clinical studies. The frequency and extent of study monitoring is defined in a monitor plan, which has been approved by the Board of Directors of the Radboudumc.

### Adverse event reporting

Adverse events are defined as any undesirable experience occurring to a participant during the study, whether or not related to the mindfulness intervention. All adverse events (AE) reported spontaneously by the subject or observed by the investigators during lab visits will be recorded in CastorEDC. AEs will be followed until they have ended, or until a stable situation has been reached. Depending on the event, follow up may require additional tests or medical procedures, and/or referral to the general physician or a medical specialist. Serious AEs (SAEs) will be reported during the full duration of the study, and will be reported through the web portal *ToetsingOnline* to the accredited medical ethical committee following national regulations. We will do so within 7 days of our first knowledge of SAEs that result in death, or for SAEs that are life threatening. After reporting, we will submit a preliminary report within 8 days. All other SAEs will be reported within 15 days after our first knowledge of the SAE.

### Communicating protocol amendments to relevant parties

All amendments shall be promptly communicated to the accredited medical ethical committee. Non-substantial amendments will be duly recorded and filed by the investigator without separate notification. Substantial amendments will not be implemented until approval of the accredited medical ethical committee has been obtained. Participants enrolled in the study will be informed in case there are changes or additions to the protocol, which might significantly impact their decision to continue participation. In this case, a new informed consent procedure will be initiated.

### Dissemination plans

Anonymized group-level results will be published in peer-reviewed national and international journals. Authorship of publications coming from this study will follow the research code: publications are submitted only with authors who have made a substantial contribution to the research. Statistical code used for any journal publications will be accessible online. Lay-friendly outcomes will be communicated to participants through newsletters, and with the broader patient population through the *Parkinson Vereniging* (the national patient association) and the ParkinsonNEXT platform. Professionals working with people with PD will be informed about the results of the study through ParkinsonNet, PD patient associations, presentations at national and international conferences, and via social media.

## Discussion

The MIND-PD trial is a prospective RCT investigating the short and long-term clinical and biological effects of MBCT in people with PD. The primary goal of this trial is to identify whether MBCT can effectively reduce symptoms of depression and anxiety in people with PD, as compared to care as usual. Beyond that, we aim to explore the effects of MBCT on: (a) *motor symptom severity*, (b) *stress biomarkers*, such as cortisol (in hair and saliva) and stress-related brain activity (fMRI before and after a strong stressor: socially evaluated cold pressor test), and (c) *PD progression biomarkers*, such as structural integrity of the substantia nigra and locus coeruleus (neuromelanin-sensitive MRI, diffusion MRI). Finally, we will explore the mechanisms underlying the effects of chronic and acute stress on PD symptoms (e.g. tremor, using accelerometry), by means of the clinical, neuroimaging and biochemical assessments at baseline. Insight into the mechanisms underlying stress and stress reduction in PD may pave the way to new treatment development.

MIND-PD adopts a large-scale and multidisciplinary approach, including psychological, neurological and neuroscientific assessments. Also, MBCT is a well-documented intervention, which, if proven to be effective, can be implemented readily on a broad scale. Other strengths of this trial include the large sample, statistically powered to detect a clinically meaningful difference on our primary endpoint. Also, we are the first to explore long term (> 3 months) effects of an MBI in PD, which allows us to not only identify relevant symptomatic effects of the intervention, but to begin to explore possible disease modifying effects as well [7]. Comprehensive outcome measures, including state of the art neuroimaging and markers of the biological stress response, will further provide unique insights into the cerebral and biochemical mechanisms of stress (reduction) in PD, which are unknown to date [7].

Potential challenges of this trial may be the risk of drop-out, due to the long (12 months) follow-up period. Also, participants and researchers are not blinded in the current study design, which means that we will not be able to rule out any expectation biases or observer-expectancy effects for certain assessments. In addition, including only individuals with mild-moderate symptoms of stress may limit the generalizability of our findings to the broader PD population. However, we expect this subgroup to benefit most from MBCT, thus providing insights that closely align with clinical practice. Furthermore, lifestyle changes (e.g. exercise, sleep) may occur simultaneously, or even as a result of mindfulness practice, and significant life events may come about, given the prospective nature of the trial. To account for potential effects of such changes, participants and their lifestyle will be monitored regularly. Effects of life events are equally expected in both groups, given the randomized design. Lastly, the passive control group implemented in this trial will allow us to draw excellent conclusions about the effects of stress reduction in PD, assuming that the applied intervention is successful. However, it will be difficult to differentiate specific from non-specific intervention effects, such as social support or attention, which may be larger in the MBCT condition [8]. We have carefully considered this point, and have opted for a passive instead of an active control (e.g. muscle stretching, or education about stress), because at this stage, we are specifically interested in the effects of stress reduction on PD, not so much in stress reduction by MBCT specifically. It is likely that active control interventions also influence stress levels in PD, which would in turn reduce the contrast between groups and thereby hinder possible conclusions about stress reduction in PD. Furthermore, much larger samples are necessary if an active control is used [10], which is not feasible given our comprehensive design with multiple cerebral and biochemical outcome measures.

To date, there are no known treatments to cure or effectively attenuate the progression of PD. In this trial, we investigate whether MBCT is effective in alleviating stress, and we take the first steps to explore whether a stress reducing intervention may perhaps provoke disease modifying effects. Short-term as well as long-term effects of MBCT will be measured with clinical, biochemical and neuroimaging assessments.

### Trial status

The study protocol described here has the following version numbers: Algemeen Beoordelings-en Registratieformulier number NL81309.091.22, and Commissie Mensgebonden Onderzoek Oost-Nederland (institutional review board) number 2022–15931, version 5 (January 9, 2024). The first participant for this study was included on April 17, 2023. As of June 3rd 2024, 43 participants have been randomized, 5 of these participants have finished all study procedures. Recruitment is expected to be completed by August 2025.

### Electronic supplementary material

Below is the link to the electronic supplementary material.

Supplementary Material 1

## Data Availability

No datasets were generated or analysed during the current study.

## Abbreviations

PD: Parkinson’s disease
RCT: Randomized controlled trial
MBCT: Mindfulness-based cognitive therapy
(f)MRI: (Functional) magnetic resonance imaging
HADS: Hospital anxiety and depression scale
MBI: Mindfulness-based intervention
CAU: Care as usual
DCCN: Donders centre for cognitive neuroimaging
Radboudumc: Radboud university medical center
MDS-UPDRS: Movement disorders society unified parkinson disease rating scale
MoCA: Montreal cognitive assessment
PSS: Perceived stress scale
PSQ-39: Parkinson’s disease questionnaire 39
PASS: Positive appraisal scale
RRS: Ruminative response scale
FFMQ: Five facet mindfulness questionnaire
GSES: General self-efficacy scale
SCS-SF: Self-compassion scale-short form
MAQ: Mindfulness adherence questionnaire
SECPT: Socially evaluated cold pressor task
RLD: Radboudumc laboratory for diagnostics
hsCRP: High sensitive c-reactive protein
TR: Repetition time
TE: Echo time
FA: Flip angle
TI: Inversion time
FOV: Field of view
LMM: Linear mixed model
FSL: FMRIB software library
SPM: Statistical parametric mapping
ICA: Independent component analysis
AE: Adverse event
SAE: Serious adverse event

## References

[1] Dorsey ER, Bloem BR (2018). The Parkinson Pandemic—A call to action. JAMA Neurol.

[2] Kish SJ, Shannak K, Hornykiewicz O (1988). Uneven pattern of dopamine loss in the striatum of patients with idiopathic Parkinson’s Disease. N Engl J Med.

[3] Hemmerle AM, Herman JP, Seroogy KB (2012). Stress, depression and Parkinson’s disease. Exp Neurol.

[4] van der Heide A, Speckens AEM, Meinders MJ, Rosenthal LS, Bloem BR, Helmich RC (2021). Stress and mindfulness in Parkinson’s disease – a survey in 5000 patients. NPJ Parkinsons Dis.

[5] de Pablos RM, Herrera AJ, Espinosa-Oliva AM, Sarmiento M, Muñoz MF, Machado A (2014). Chronic stress enhances microglia activation and exacerbates death of nigral dopaminergic neurons under conditions of inflammation. J Neuroinflammation.

[6] Burtscher J, Copin J-C, Rodrigues J, Kumar ST, Chiki A, Guillot de Suduiraut I et al. Chronic corticosterone aggravates behavioral and neuronal symptomatology in a mouse model of alpha-synuclein pathology. Neurobiol Aging. 2019;83;Pre-press:11–20. 10.1016/j.neurobiolaging.2019.08.007 31585362

[7] Goltz F, van der Heide A, Helmich RC. Alleviating stress in Parkinson’s Disease: symptomatic treatment, Disease Modification, or both? J Parkinsons Dis. 2024;1–12. 10.3233/JPD-230211 PMC11380242 38363618

[8] van der Heide A, Meinders MJ, Speckens AEM, Peerbolte TF, Bloem BR, Helmich RC (2021). Stress and mindfulness in Parkinson’s Disease: clinical effects and potential underlying mechanisms. Mov Disord.

[9] Kabat-Zinn J (2013). Full catastrophe living, revised edition: how to cope with stress, pain and illness using mindfulness meditation.

[10] Goldberg SB, Riordan KM, Sun S, Davidson RJ (2022). The empirical status of mindfulness-based interventions: a systematic review of 44 Meta-analyses of Randomized controlled trials. Perspect Psychol Sci.

[11] Nauta IM, van Dam M, Bertens D, Kessels RPC, Fasotti L, Uitdehaag BMJ (2024). Improved quality of life and psychological symptoms following mindfulness and cognitive rehabilitation in multiple sclerosis and their mediating role for cognition: a randomized controlled trial. J Neurol.

[12] Dissanayaka NNW, Idu Jion F, Pachana NA, O’Sullivan JD, Marsh R, Byrne GJ (2016). Mindfulness for Motor and Nonmotor dysfunctions in Parkinson’s Disease. Parkinsons Dis.

[13] Kwok JYY, Kwan JCY, Auyeung M, Mok VCT, Lau CKY, Choi KC (2019). Effects of Mindfulness yoga vs stretching and resistance training exercises on anxiety and depression for people with Parkinson Disease. JAMA Neurol.

[14] Rodgers SH, Schütze R, Gasson N, Anderson RA, Kane RT, Starkstein S (2019). Modified mindfulness-based cognitive therapy for depressive symptoms in Parkinson’s Disease: a pilot trial. Behav Cogn Psychother.

[15] Pickut BA, Van Hecke W, Kerckhofs E, Mariën P, Vanneste S, Cras P (2013). Mindfulness based intervention in Parkinson’s disease leads to structural brain changes on MRI. Clin Neurol Neurosurg.

[16] Kwok JYY, Choi EPH, Wong JYH, Lok KYW, Ho M-H, Fong DYT (2023). A randomized clinical trial of mindfulness meditation versus exercise in Parkinson’s disease during social unrest. NPJ Parkinsons Dis.

[17] Pickut B, Vanneste S, Hirsch MA, Van Hecke W, Kerckhofs E, Mariën P (2015). Mindfulness training among individuals with Parkinson’s Disease: Neurobehavioral effects. Parkinsons Dis.

[18] Postuma RB, Berg D, Stern M, Poewe W, Olanow CW, Oertel W (2015). MDS clinical diagnostic criteria for Parkinson’s disease. Mov Disord.

[19] Segal A, Williams J, Teasdale J. Mindfulness-Based Cognitive Therapy for Depression. 2nd edition. New York: Guilford Press; 2018.

[20] British Association of Mindfulness-based Approaches. Good Practice Guidelines for Teaching Mindfulness-Based Courses. https://bamba.org.uk/good-practice-guidelines/.

[21] Crane RS, Eames C, Kuyken W, Hastings RP, Williams JMG, Bartley T (2013). Development and validation of the mindfulness-based interventions – Teaching Assessment Criteria (MBI:TAC). Assessment.

[22] Hassed C, Flighty A, Chambers R, Hosemans D, Bailey N, Connaughton S (2021). Advancing the Assessment of Mindfulness-based meditation practice: psychometric evaluation of the Mindfulness Adherence Questionnaire. Cognit Ther Res.

[23] Bouwmans C, De Jong K, Timman R, Zijlstra-Vlasveld M, Van der Feltz-Cornelis C, Tan SS (2013). Feasibility, reliability and validity of a questionnaire on healthcare consumption and productivity loss in patients with a psychiatric disorder (TiC-P). BMC Health Serv Res.

[24] Rodriguez-Blazquez C, Frades‐Payo B, Forjaz MJ, de Pedro‐Cuesta J, Martinez‐Martin P (2009). Psychometric attributes of the hospital anxiety and Depression Scale in Parkinson’s disease. Mov Disord.

[25] ter Avest MM, van Velthoven ASM, Speckens AEM, Dijkstra G, Dresler M, Horjus CS (2023). Effectiveness of mindfulness-based cognitive therapy in reducing psychological distress and improving sleep in patients with inflammatory bowel disease: study protocol for a multicentre randomised controlled trial (MindIBD). BMC Psychol.

[26] Compen F, Bisseling E, Schellekens M, Donders R, Carlson L, van der Lee M (2018). Face-to-face and internet-based mindfulness-based cognitive therapy compared with treatment as Usual in reducing psychological distress in patients with Cancer: a Multicenter Randomized Controlled Trial. J Clin Oncol.

[27] Spinhoven PH, Ormel J, Sloekers PPA, Kempen GIJM, Speckens AEM, van Hemert AM (1997). A validation study of the hospital anxiety and Depression Scale (HADS) in different groups of Dutch subjects. Psychol Med.

[28] Goetz CG, Tilley BC, Shaftman SR, Stebbins GT, Fahn S, Martinez-Martin P (2008). Movement Disorder Society‐sponsored revision of the Unified Parkinson’s Disease Rating Scale (MDS‐UPDRS): Scale presentation and clinimetric testing results. Mov Disord.

[29] Zach H, Dirkx MF, Roth D, Pasman JW, Bloem BR, Helmich RC (2020). Dopamine-responsive and dopamine-resistant resting tremor in Parkinson disease. Neurology.

[30] Dalrymple-Alford JC, MacAskill MR, Nakas CT, Livingston L, Graham C, Crucian GP (2010). The MoCA. Neurology.

[31] Bruijnen CJWH, Dijkstra BAG, Walvoort SJW, Budy MJJ, Beurmanjer H, De Jong CAJ (2020). Psychometric properties of the Montreal Cognitive Assessment (MoCA) in healthy participants aged 18–70. Int J Psychiatry Clin Pract.

[32] Ligneul R, Mainen ZF, Ly V, Cools R (2022). Stress-sensitive inference of task controllability. Nat Hum Behav.

[33] Schwabe L, Haddad L, Schachinger H (2008). HPA axis activation by a socially evaluated cold-pressor test. Psychoneuroendocrinology.

[34] Cohen S. Perceived stress in a probability sample of the United States. In: Spacapan S, Oskamp S, editors. The social psychology of health. Sage Publications, Inc.; 1988. pp. 31–67.

[35] Treynor W, Gonzalez R, Nolen-hoeksema S (2003). Rumination reconsidered: a psychometric analysis. Cognit Ther Res.

[36] Raes F, Pommier E, Neff KD, Van Gucht D (2011). Construction and factorial validation of a short form of the Self-Compassion Scale. Clin Psychol Psychother.

[37] Nilsson M, Hagell P, Iwarsson S (2015). Psychometric properties of the General Self-Efficacy Scale in Parkinson’s disease. Acta Neurol Scand.

[38] Schwarzer R, Jerusalem M. General Self-Efficacy Scale (GSE). APA PsycTests. 1995.

[39] van der Heide A, Dommershuijsen LJ, Puhlmann LMC, Kalisch R, Bloem BR, Speckens AEM (2024). Predictors of stress resilience in Parkinson’s disease and associations with symptom progression. NPJ Parkinsons Dis.

[40] Petri-Romão P, Engen H, Rupanova A, Puhlmann L, Zerban M, Neumann RJ (2024). Self-report assessment of positive Appraisal Style (PAS): development of a process-focused and a content-focused questionnaire for use in mental health and resilience research. PLoS ONE.

[41] Baer RA, Smith GT, Lykins E, Button D, Krietemeyer J, Sauer S (2008). Construct validity of the five Facet Mindfulness Questionnaire in Meditating and Nonmeditating samples. Assessment.

[42] Bohlmeijer E, ten Klooster PM, Fledderus M, Veehof M, Baer R (2011). Psychometric properties of the five facet mindfulness questionnaire in depressed adults and development of a short form. Assessment.

[43] Kliem S, Mößle T, Rehbein F, Hellmann DF, Zenger M, Brähler E (2015). A brief form of the Perceived Social Support Questionnaire (F-SozU) was developed, validated, and standardized. J Clin Epidemiol.

[44] Marinus J, Visser M, Jenkinson C, Stiggelbout AM (2008). Evaluation of the Dutch version of the Parkinson’s Disease Questionnaire 39. Parkinsonism Relat Disord.

[45] Borm GF, Fransen J, Lemmens WAJG (2007). A simple sample size formula for analysis of covariance in randomized clinical trials. J Clin Epidemiol.

[46] Johansson ME, Cameron IGM, Van der Kolk NM, Vries NM, Klimars E, Toni I (2022). Aerobic Exercise alters brain function and structure in Parkinson’s Disease: a Randomized Controlled Trial. Ann Neurol.

[47] Vijiaratnam N, Foltynie T (2023). How should we be using biomarkers in trials of disease modification in Parkinson’s disease?. Brain.

[48] Pruessner JC, Kirschbaum C, Meinlschmid G, Hellhammer DH (2003). Two formulas for computation of the area under the curve represent measures of total hormone concentration versus time-dependent change. Psychoneuroendocrinology.

[49] Pasternak O, Sochen N, Gur Y, Intrator N, Assaf Y (2009). Free water elimination and mapping from diffusion MRI. Magn Reson Med.

[50] Beckmann C, Mackay C, Filippini N, Smith S (2009). Group comparison of resting-state FMRI data using multi-subject ICA and dual regression. NeuroImage.

[51] Smith SM, Fox PT, Miller KL, Glahn DC, Fox PM, Mackay CE (2009). Correspondence of the brain’s functional architecture during activation and rest. Proc Natl Acad Sci U S A.

